# Baicalein ameliorates DSS-induced ulcerative colitis in mice by inhibiting ferroptosis and regulating gut microbiota

**DOI:** 10.3389/fphar.2025.1564783

**Published:** 2025-07-31

**Authors:** Weiguang Lv, Shengnan Han, Ke Li, Aimin Yan, Wei Wang, Wanping Lu, Jing Han, Chenggang Zhang

**Affiliations:** School of Life Sciences, Beijing University of Chinese Medicine, Beijing, China

**Keywords:** ulcerative colitis, baicalein, oxidative stress, inflammation, ferroptosis, gut microbiota

## Abstract

Ulcerative colitis (UC) is a nonspecific inflammatory disease. Baicalein has potential value in treating UC, but its mechanism is unclear. This study aims to evaluate the protective effects of baicalein on dextran sodium sulfate (DSS)-induced UC mice. The UC model was established by 4% DSS solution for 7 days. Treatments included baicalein (10 mg/kg, 20 mg/kg) and sulfasalazine (200 mg/kg) via oral gavage. Colonic damage was assessed through body weight, disease activity index (DAI), histopathology (H&E staining) and colon length. Inflammatory cytokines were measured by ELISA, while oxidative stress markers and iron content were analyzed by colorimetric assays. Protein expression was evaluated by Western blot, and gene levels by RT-qPCR. Intestinal microbiota changes were characterized using 16S rRNA gene sequencing. Results demonstrated that Baicalein ameliorated UC mice, particularly in high-dose of baicalein group. After baicalein treatment, the proinflammatory cytokines (TNF-α, IL-1β), and anti-inflammatory cytokine (IL-10) has decreased. Additionally, high-dose of baicalein strongly reversed oxidative stress alterations caused by DSS, as evidenced by Fe^2+^, MDA, ROS significantly depleted, and MPO, SOD, GSH significantly increased. Protein and mRNA expression analyses revealed that high-dose baicalein upregulated the expression of FTH1, GPX4, SLC7A11, SLC3A2 and Nrf2, while downregulating ACSL4 significantly. Microbiological analysis showed that baicalein ameliorated intestinal dysbiosis, increased Ligilactobacillus and NK4A136, while reduced *Clostridium*_sensu_stricto_1 and Escherichia-Shigella. These findings suggest that baicalein mitigates DSS-induced UC mice by reducing oxidative stress and inflammation, suppressing ferroptosis and modulating gut microbiota composition, Proposing a potentially effective therapeutic approach for UC.

## 1 Introduction

Ulcerative colitis (UC) is a prevalent form of inflammatory bowel disease (IBD) characterized by chronic inflammatory colonic mucosa. Rectal blood, diarrhea, bowel urgency, fecal incontinence, and bouts of cramping stomach pain are all signs of UC (Baumgart and Sandborn, 2007; Seyedian et al., 2019). The pathophysiology of UC remains unclear but is associated with genetic factors, immune responses, oxidative stress, and microbial infections (de Souza et al., 2017). Currently, medications for UC include serious adverse effects such high recurrence, fever, and infection risk. Recent research suggests that targeting different types of cell death, such as ferroptosis (Kerr et al., 1972), cellular autophagy (Eskelinen and Saftig, 2009), and cysteine-dependent apoptosis (Stockwell et al., 2017), may offer novel therapeutic approaches for UC.

Ferroptosis is characterized by the generation of reactive oxygen species (ROS) through excess iron-induced peroxidation of polyunsaturated fatty acids (PUFA), leading to membrane damage, rupture, and eventual cell death (Dixon et al., 2012). The metabolic pathway of ferroptosis involves glutathione (GSH) depletion or Glutathione Peroxidase 4 (GPX4), in conjunction with iron-catalyzed production of lipid free radicals (Linkermann et al., 2014). Activation of nuclear factor erythropoietin-2-related factor 2 (Nrf2) upregulates antioxidant genes such as GPX4 and GSH, offering protection against iron accumulation and oxidative stress (Wu et al., 2021). Solute carrier family 7 member 11 (SLC7A11), involved in amino acid transport across the plasma membrane, forms a disulfide bond with solute carrier family 3 member 2 (SLC3A2), impacting the expression of GXP4 by modulating cellular cysteine levels and influencing lipid peroxidation in the cell membrane (Cao and Dixon, 2016). Additionally, acyl coenzyme A synthetase long-chain family member 4 (ACSL4) enhances lipid peroxide production (Doll et al., 2017) facilitates PUFA esterification to acyl-CoA both contributing to ferroptotic cell death (Kagan et al., 2017).

With the development of sequencing technologies, the link between UC and gut microorganisms is becoming increasingly clear (Luo et al., 2024; Xiong et al., 2022). Host metabolism and immune response are influenced by microorganisms and their metabolites (Ruff et al., 2020). On the one hand, inflammatory responses and oxidative stress in the body are triggered by alterations in the composition and quantity of gut flora. For example, an increase in the phylum Anaplasma and *Clostridium* often increases the body’s anti-inflammatory capacity (Guo et al., 2020; Maldonado-Arriaga et al., 2021), while an increase in the Firmicutes and Ascomycetes often triggers an inflammatory response and the onset of oxidative stress (Kelly et al., 2021; Loi et al., 2023; Vitetta et al., 2017). On the other hand, a reduction in bacterial variety and an breakdown in the dominant flora (including Firmicutes, anamorphs, actinomycetes and ascomycetes) can lead to lipid peroxidation of cell membranes, causing ferroptosis and ultimately the development of UC (Elinav et al., 2011; Ma et al., 2022).

Scutellaria baicalensis Georgi, a widely recognized herbal remedy with a complex composition, has been utilized in traditional Chinese medicine for millennia. Baicalein, the principal flavonoid monomer in Scutellaria baicalensis Georgi, undergoes metabolic transformation and absorption facilitated by intestinal microorganisms, thereby manifesting a diverse array of pharmacological properties such as anti-inflammatory, antimicrobial, and antioxidant effects (Li Y. Y. et al., 2022). Recent studies have elucidated that baicalein ameliorates acute liver injury in murine models by activating the Nrf2 pathway while concurrently suppressing the NF-κB pathway (Dai et al., 2021). Lai et al. observed a dose-dependent downregulation of GPX4 expression by baicalein through the JAK2/STAT3/GPX4 pathway in cell, leading to elevated levels of ROS and malondialdehyde (MDA), culminating in ferroptotic cell death in colorectal cancer cells (Lai et al., 2024). Despite extensive studies on the impact of baicalein in various conditions such as neoplastic diseases, female reproductive disorders (Li et al., 2024) and renal diseases (Guo et al., 2024), research on its role in ferroptosis in UC remains limited. Therefore, this study aims to investigate the modulatory effects of baicalein on the gut microbiota of mice with UC, as well as its influence on iron-induced oxidative stress and the ferroptosis pathway.

## 2 Materials and methods

### 2.1 Chemicals and reagents

Baicalein (CAS: 491-67-8) was obtained from Yuanye Biotechnology Co., Ltd (Shanghai, China). Sodium Dextran Sulfate 5,000 (CAS: 199–08361) was purchased from FUJIFILM Wako Pure Chemical Co., Ltd (Japan). Sulfasalazine tablets (SASP) enteric-coated tablets (CAS: H31020840) was purchased from Shanghai Fuda Pharmaceutical Company. Sodium cellulose acetate (CAS: 9004-35-7) was obtained from SapoRex Technology Co., Ltd (Shanghai, China). Primary antibodies: ferritin heavy chain 1 (FTH1) (CAS: 3998S), GPX4 (CAS: 52455S), SLC7A11 (CAS: 12691S), SLC3A2 (CAS: 47213T), Nrf2 (CAS:12721T) were obtained from Cell Signaling Technology Co., Ltd (United States). ACSLA4/FACL4 (CAS: 155,282) was purchased from Abcam Co., Ltd (United States); β-actin (CAS: 9004-35-7) as internal reference was obtained from Sapphirex Co., Ltd (Beijing, China). HRP* Goat Anti Mouse IgG (RS0001) and HRP* Goat Anti Rabbit IgG (RS0002) were purchased from ImmunoWay Biotechnology Co., Ltd. (United States). The 4x SDS Protein Sampling Buffer (G2526-1), Tris-MOPS-SDS Instant Pellet (T7205M), Transmembrane Buffer Instant Pellet (Z0202M), TBST Instant Pellet (7.4 ± 0.15@25s°C) (T7209M) and pre-stained Protein Marker 10-180KD (P1018-250 μL) were purchased from Beijing Lamblade Trading Co., Ltd (Beijing, China). Ultrasensitive ECL chemiluminescence kit (NcmECL Ultra) (P10100) was purchased from Suzhou New Saimei Biotechnology Co., Ltd (Suzhou, China).

### 2.2 Animal

Male specific pathogen-free (SPF) C57BL/6J mice (*N* = 50, 6–8 weeks old, 18–22 g) were obtained from SPF Biotechnology Co. Ltd. (License No. SCXK 2024-0001, Beijing, China). Animals were housed in a controlled laboratory environment (temperature: 25°C ± 1°C, humidity: 50%–70%) at the Animal Experimentation Center of Beijing University of Chinese Medicine (BUCM). All experimental procedures were conducted in accordance with BUCM Guidelines for Laboratory Animal Use and approved by both the Animal Teaching and Research Committee and Medical Ethics Committee of BUCM (Ethics No. BUCM-2023061901-2164). All experimental procedures were performed by certified personnel. Euthanasia was conducted under isoflurane anesthesia, with predefined humane endpoints (including but not limited to: ≥20% body weight loss, severe diarrhea, or mobility impairment) implemented to minimize animal suffering.

**FIGURE 1:**
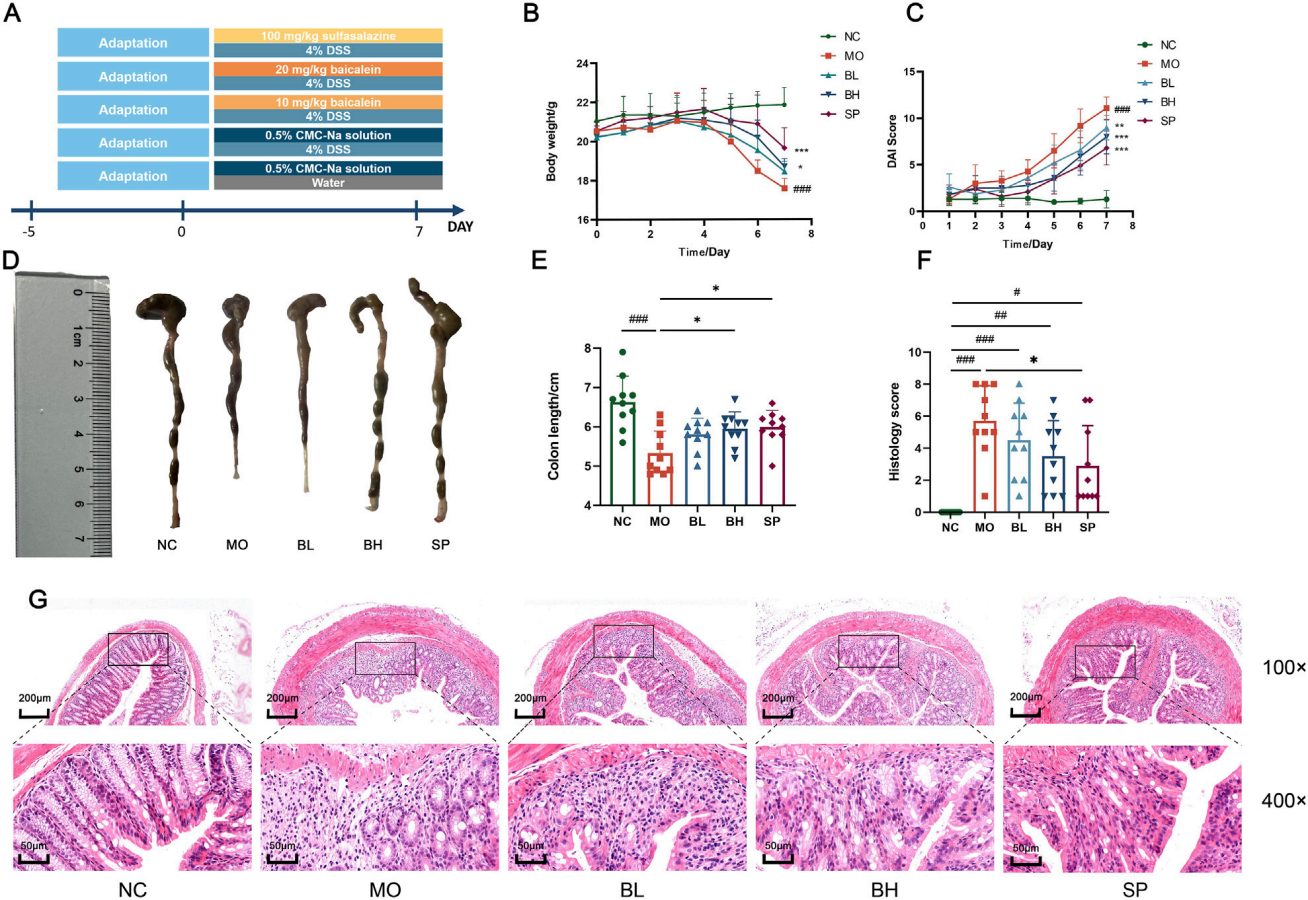
Baicalein exerts a protective effect on the DSS-induced acute UC model of mice. **(A)** Establishment of the UC model and drug intervention. **(B)** Body weight changes in mice. **(C)** DAI scores in mice. **(D)** Colon length images. **(E)** Measurement of colon length. **(F)** Histopathological scoring of colon tissues. **(G)** Photographs of H&E staining of colon tissues. (Data were analyzed by one-way ANOVA and Kruskal–Wallis test, with results presented as means ± *SD*. *N* = 10. ^#^ *p* < 0.05, ^##^ *p* < 0.01, ^###^ *p* < 0.001 vs. NC group; ^*^ *p* < 0.05, ^**^ *p* < 0.01, ^***^ *p* < 0.001 vs. MO group).

After 5 days of acclimatization, mice were randomly divided into 5 groups (*N* = 10 for each group): normal control (NC) group, model (MO) group, low-dose of baicalein (BL) group, high-dose of baicalein (BH) group, and sulfasalazine (SP) group. As illustrated in Figure 1A, the acute UC model was initiated by 4% (w/v) DSS solution, except for the NC group, which received sterile water. The BL, BH, and SP groups were treated with 10 mg/kg and 20 mg/kg baicalein, and 100 mg/kg sulfasalazine by gavage, respectively. Both control and experimental groups received 0.5% solution of Carboxymethyl Cellulose Sodium (CMC)-Na by gavage once daily for 7 consecutive days. All experimental animals received standard laboratory diet during the study period.

Blood samples were collected on day 8 after a 24-hour fast from the orbital sinus. Euthanasia was performed through cervical dislocation. The colon was excised promptly, measured, and rinsed with saline. It was then divided into three segments, with the distal part preserved in 4% paraformaldehyde. Both the colon and serum were promptly stored at −80°C for subsequent analysis.

### 2.3 DAI score

During medication treatment, mice were observed daily for alterations in body weight, food consumption, physical activity, behavior, stool consistency, and fecal blood. The DAI score was determined by averaging three parameters: body weight reduction, fecal blood, and stool consistency (DAI = Σ scores/3) following reference (Wang et al., 2022).

### 2.4 Histological analysis

The colon tissues were fixed with 4% paraformaldehyde and subsequently subjected to H&E staining. The specimens were processed through dehydration, paraffin embedding, sectioning, deparaffinization, and hematoxylin staining, differentiated for color separation, stained with eosin, dehydrated, cleared, and sealed. Subsequently, the samples were subjected to histopathological analysis using a super-resolution microscope, assessing inflammatory cell infiltration and epithelial cell morphology in the mucosa.

### 2.5 ELISA analysis

Colon tissues were homogenized in phosphate buffer (1:9, w/v) using a tissue grinder. And centrifuge (3,000 r/min, 10 min, 4°C) to obtain the extract for cytokine analysis. According to the instructions, TNF-α, IL-1β, and IL-10 levels were assessed following the ELISA kits (Cat: No. SEKM-0034, SEKM-0002, and SEKM-001). The concentrations were determined using four-parameter logistic regression analysis.

### 2.6 Oxidative stress and detection of iron levels

Total protein concentration in colonic homogenates was measured using a BCA Protein Assay Kit (B5001). The levels of MPO, MDA, SOD, ROS, GSH and iron were quantified using commercial biochemical assay kits (Nanjing Jiancheng, China) according to manufacturer’s specifications.

### 2.7 Western blot

Protein levels of FTH1, GPX4, SLC7A11, SLC3A2, ACSL4, and Nrf2 in mice colon tissues were analyzed by Western blot. Colon tissues were removed from −80°C storage, shred, and added to RIPA lysate containing protease (C0101-1) and phosphatase inhibitors (C0104). The mixture was lysed on ice to extract proteins. Tissue samples were centrifuged (12,000 rpm, 30 min, 4 °C) to determined protein concentrations using the BCA kit. Protein extracts were denatured in 4 × loading buffer at 100°C for 5 min.

Proteins were loaded into the 10% SDS-PAGE using a One-Step PAGE Gel Rapid Preparation Kit (038411800) and transferred to PVDF membranes. Membranes were blocked in 5% skim milk in TBST at room temperature for 2 h, and incubated at 4°C overnight with the primary antibody. Then incubated with secondary antibodies at room temperature for 1 h. Protein bands were imaged through ECL reagent and captured using a gel imaging system (Tannon, 5,200). Gray values were analyzed using ImageJ 1.54, with β-actin serving as internal control.

### 2.8 Quantitative reverse transcription PCR

RNA from colon tissue was extracted using the RNA extraction kit (AG21017). The NanoDrop 2000C spectrophotometer assessed RNA concentration and quality. EvoM-MLV Reverse Transcription Reagent Pre-mix (AG11706) was used to reverse transcription of total RNA into cDNA. The genes including FTH1, GPX4, SLC7A11, SLC3A2, ACSL4 and Nrf2 were analyzed by SYBR Green PCR in QuantStudio™ Real-Time PCR system. Relative expression of genes levels were analyzed by 2^−ΔΔCT^ method. Gene-specific primer sequences are listed in Table 1.

**TABLE 1:** The primer sequences list.

Gene name	Fragment size (bp)	Forward primer (5′-3′)	Reverse primer (5′-3′)
β-Actin	140	AATCGTGCGTGACATCAA	GCTCGTTGCCAATAGTGA
Nrf2	149	GCA​CAT​CCA​GAC​AGA​CAC​CAG	TAT​CCA​GGG​CAA​GCG​ACT​CAT
GPX4	83	CCC​GAT​ATG​CTG​AGT​GTG​GTT​TA	TTC​TTG​ATT​ACT​TCC​TGG​CTC​CTG
SLC3A2	74	ACG​GTG​TGG​ATG​GTT​TCC​AA	CTG​CCA​CTC​AGC​CAA​GTA​CA
SLC7A11	174	GAG​CCC​TGT​CCT​ATG​CAG​AA	GGA​TGT​AGC​GTC​CAA​ATG​CC
ACSL4	138	GCC​ATG​GAA​GCT​GAA​ATA​CTG​AAA​G	GAA​GGC​ATC​TGT​TAC​CAA​ACC​AGT​C
FTH1	99	CAT​CAA​CCG​CCA​GAT​CAA​CCT	GCA​AAG​TTC​TTC​AGA​GCC​ACA​TCA

### 2.9 16S rRNA analysis

Fecal specimens were collected in sterile microcentrifuge tubes and stored immediately at −80°C. Fecal genomic DNA was isolated with a DNA Isolation Kit (MoBio, Carlsbad, CA, United States) and quantified for purity and concentration. The 16S rRNA V3-V4 region was amplified with the primer 341F (5′-CCTAYGGGRBGCASCAG-3′) and 806R (5′-GGACTACNNGGGGTATCTAAT-3′). The products were analyzed for fecal bacterial content by Illumina sequencing after purification. High quality sequences with a 97% similarity threshold were classified into an OTU following quality filtering and assembly. Alpha diversity metrics were calculated based on OTU abundance and taxonomic classification. Beta diversity was evaluated with principal component analysis (PCA). Linear discriminant analysis effect size (LEfSe) was used to determine the different marker properties between groups. Functional prediction of microbial communities was analyzed by PICRUSt. Correlations between intestinal flora and inflammatory factors, oxidative stress, and ferroptosis-related proteins were analyzed using Pearson correlation by R language (version 4.2.0).

### 2.10 Statistical analysis

Data are presented as mean ± standard deviation (SD). ANOVA was used to assess significant differences between multiple groups. When one-way ANOVA was inappropriate, the non-parametric Kruskal–Wallis test was used. Additionally, when the data were normally distributed but had unequal variances, the Dunnett’s T3 test for multiple comparisons was used. A *p*-value of less than 0.05 was used as the threshold for statistical significance.

## 3 Results

### 3.1 Baicalein ameliorated DSS-induced UC in mice

To investigate the physiological functions of the gastrointestinal tract and the curative efficacy of baicalein on DSS-induced colitis mice, we assessed DAI scores and conducted H&E staining of gastrointestinal tissues. Mice in MO group showed body weight loss, diarrhea, bloody stools, significantly reduced colon length (*p* < 0.001), and higher DAI scores (*p* < 0.001) compared to normal control (NC) group (Figures 1B,C). Treatment with baicalein of different concentrations (10 mg/kg in BL group and 20 mg/kg in BH group) and sulfasalazine (SASP) (SP group) resulted in increased colon length and significantly reduced DAI scores (*p* < 0.001) (Figures 1D,E). H&E staining showed reduced crypt loss, depletion of goblet cells, epithelial damage, edema, and infiltration of inflammatory cells in the treatment group compared to the MO group (Figure 1G). H&E‐stained sections were histologically scored per standardized criteria (Table 2). Histological scores were significantly reduced in all treatment groups compared to the MO group (*p* < 0.001) (Figure 1F). Collectively, histological analysis suggests baicalein facilitates restoration of intestinal barrier function.

**TABLE 2:** The colon histological score standard.

Score	Inflammation	Intestinal mucosa
0	No	Normal mucosal architecture without edema
1	Minimal	Mild interstitial edema with superficial mucosal damage
2	Moderate	Moderate interstitial edema with mucosal and submucosal involvement
3	Severe	Severe interstitial edema with full-thickness injury

### 3.2 Baicalein reduces inflammatory and oxidative stress markers in colonic tissues of DSS-induced UC mice

**FIGURE 2:**
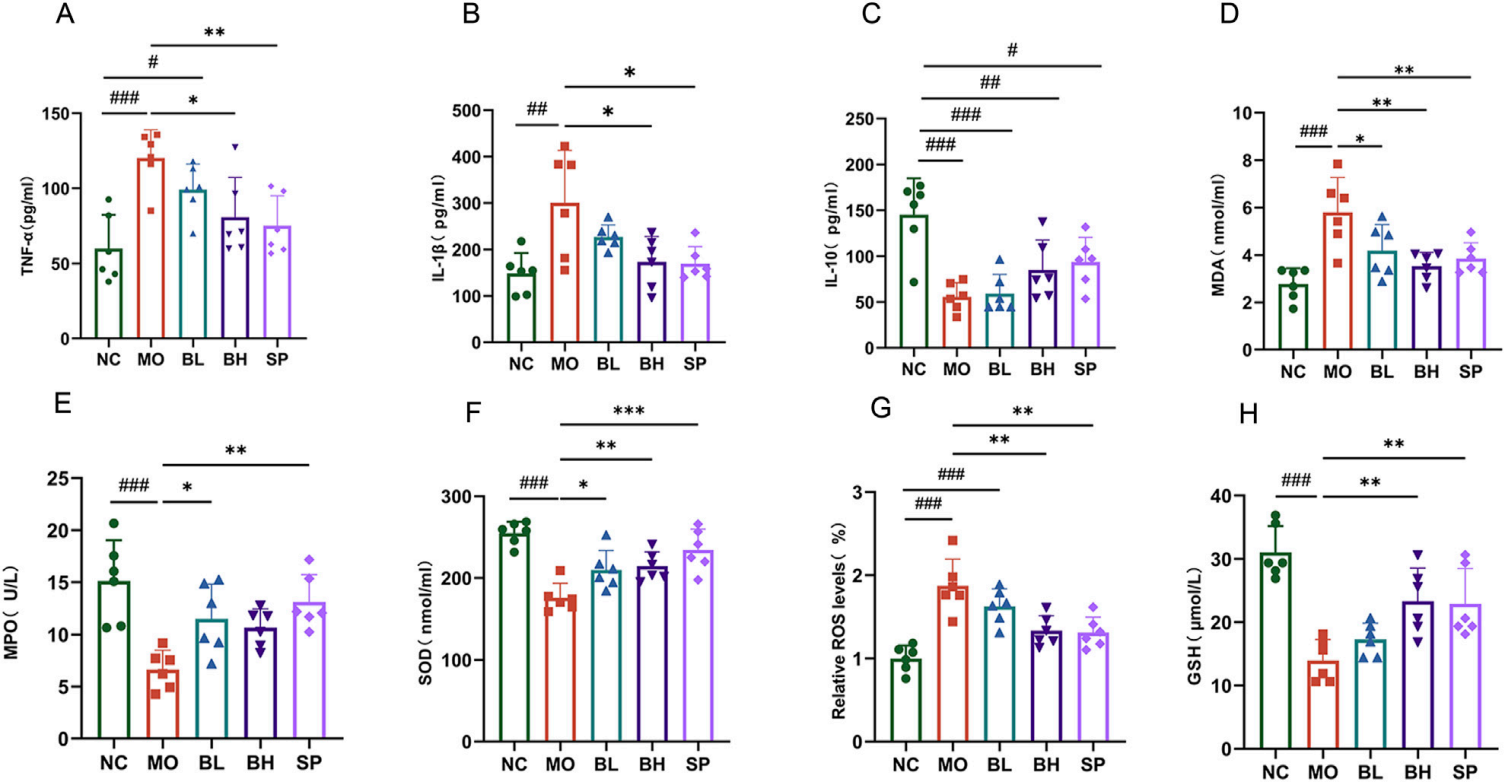
Baicalein inhibits the inflammatory response and oxidative stress in the colonic tissues of UC mice. **(A–C)** Inflammatory factor level in colon tissue: TNF-α(A), IL-1β **(B)**, IL-10 **(C)**. **(D–H)** Oxidative stress levels in colon tissue: MDA **(D)**, MPO **(E)**, SOD **(F)**, ROS **(G)**, GSH **(H)**. (Data were analyzed by one-way ANOVA, with results presented as means ± SD. *N* = 6. ^#^ *p* < 0.05, ^##^ *p* < 0.01, ^###^ *p* < 0.001 vs. NC group; ^*^ *p* < 0.05, ^**^ *p* < 0.01, ^***^ *p* < 0.001 vs. MO group).

Baicalein’s effects on oxidative stress and inflammation in DSS-induced UC mice was investigated using ELISA to measure TNF-α, IL-1β, and IL-10 levels. Biochemical kits were utilized to assess ROS, MDA, MPO, and SOD in colon tissue. The MO group exhibited significantly elevated TNF-α and IL-1β levels (*p* < 0.001) and decreased IL-10 levels compared to NC group. Following baicalein treatment, TNF-α and IL-1β levels decreased, while IL-10 levels increased. The BH group displayed more pronounced changes than BL group (Figures 2A–C). Regarding oxidative stress, MPO, SOD, and GSH levels were notably lower in MO group than in NC group (*p* < 0.001), whereas MDA levels were significantly higher (*p* < 0.001). Baicalein and sulfasalazine increased MPO, SOD, and GSH levels and decreased MDA levels in DSS-induced mice as anticipated. The BH and SP groups exhibited stronger impacts than BL group (Figures 2D–H). These findings demonstrate that baicalein diminishes oxidative stress and proinflammatory markers in DSS-induced mice with a dose-dependent manner.

### 3.3 Baicalein treatment inhibits ferroptosis in colonic tissues of DSS-induced UC in mice

**FIGURE 3:**
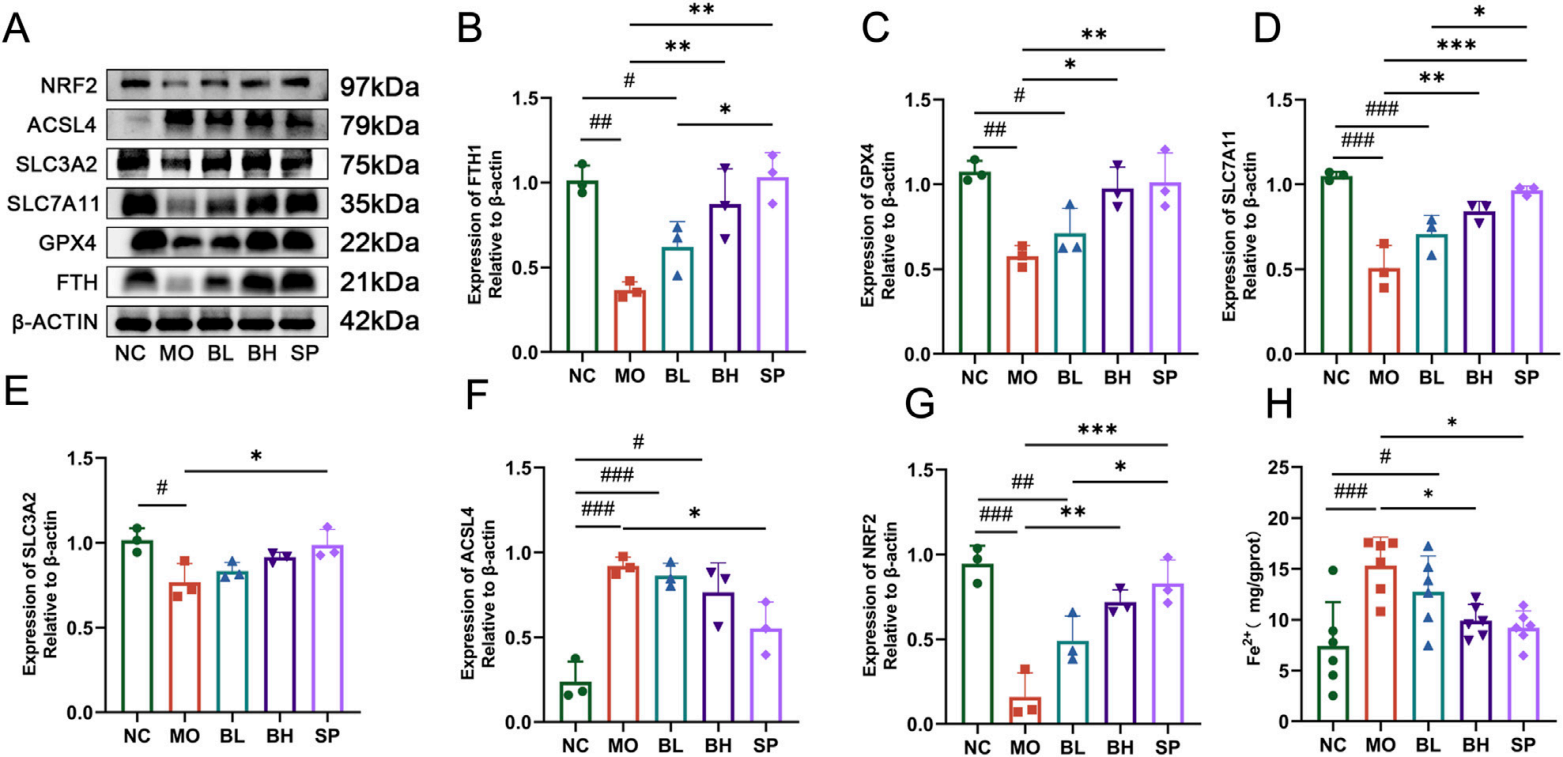
The influences of baicalein on the protein expression levels and the Fe^2+^ level in colon tissue of UC mice. **(A)** Western blot analysis of the ferroptosis-related proteins. **(B–G)** The quantification analysis of protein expression levels: FTH1 **(B)**, GPX4 **(C)**, SLC7A11 **(D)**, SLC3A2 **(F)**, ACSL4 **(E)**, Nrf2 **(G)**. **(H)** The quantification analysis of Fe^2+^ expression levels. (Data were analyzed by one-way ANOVA, with results presented as means ± SD. *N* = 6. ^#^ *p* < 0.05, ^##^ *p* < 0.01, ^###^ *p* < 0.001 vs. NC group; ^*^ *p* < 0.05, ^**^ *p* < 0.01, ^***^ *p* < 0.001 vs. MO group).

**FIGURE 4:**
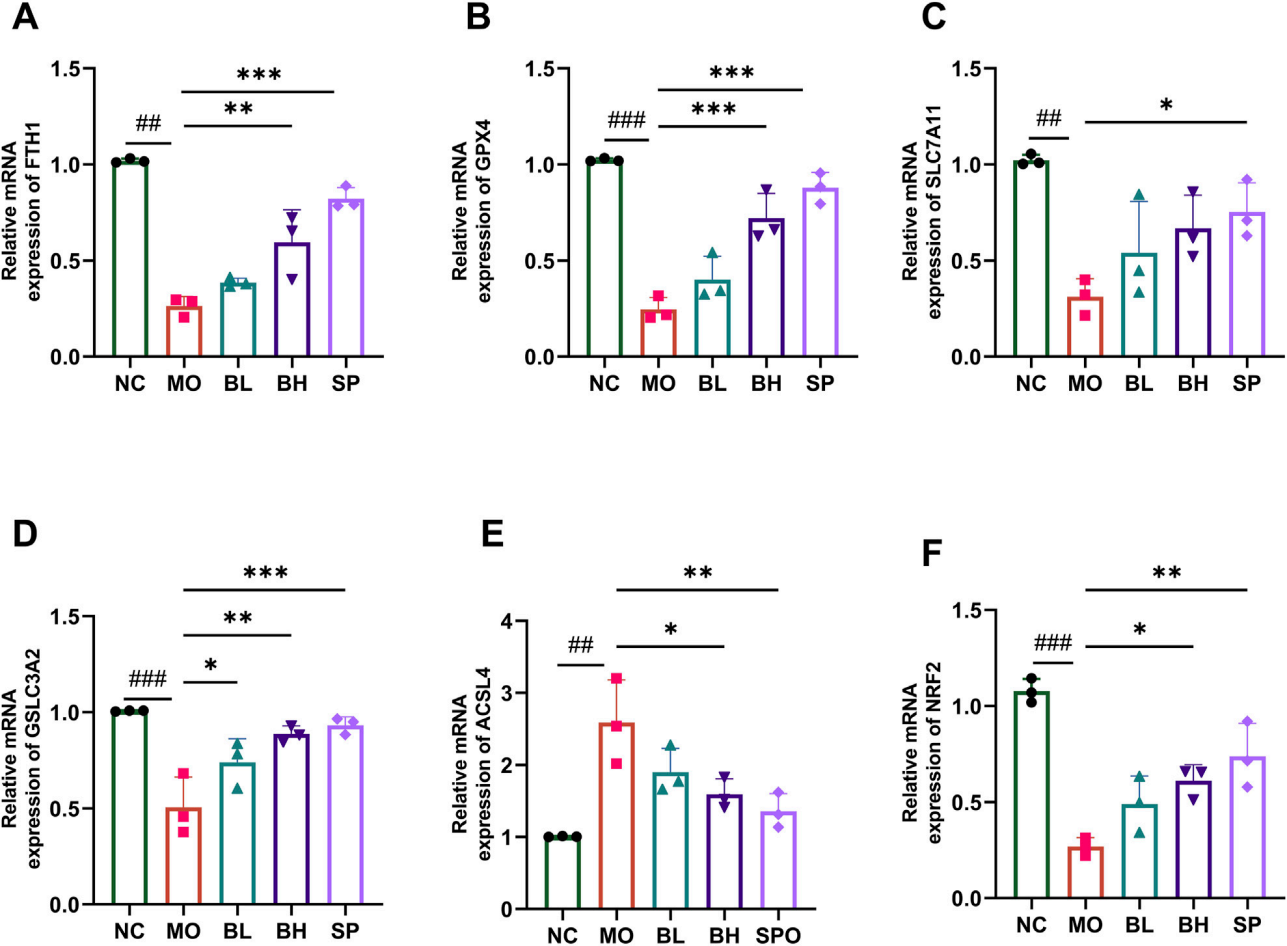
The influences of baicalein on the mRNA expression level of ferroptosis-associated genes. The mRNA expression of FTH1 **(A)**, GPX4 **(B)**, SLC7A11 **(C)**, SLC3A2 **(D)**, ACSL4 **(E)**, Nrf2 **(F)** in different groups. (Data were analyzed by one-way ANOVA, with results presented as means ± SD. *N* = 6. ^#^ *p* < 0.05, ^##^ *p* < 0.01, ^###^ *p* < 0.001 vs. NC group; ^*^ *p* < 0.05, ^**^ *p* < 0.01, ^***^ *p* < 0.001 vs. MO group).

Tissue iron detection using a biochemical kit showed a significant increase in colonic iron levels in UC mice, which decreased post baicalein intervention. The iron levels were significantly different in BH and SP groups compared to the MO group (Figure 3H). Western blotting and RT-qPCR techniques were employed to measure the protein and mRNA expression levels of critical ferroptosis pathway-related proteins, such as FTH1, GPX4, SLC7A11, SLC3A2, ACSL4, and Nrf2. Western blot analysis demonstrated significantly reduced levels of FTH1 (*p* < 0.05), GPX4 (*p* < 0.01), SLC7A11 (*p* < 0.001), SLC3A2 (*p* < 0.05), and Nrf2 (*p* < 0.001) in the MO group compared to NC group, alongside increased ACSL4 levels (*p* < 0.01). After treatment with baicalein of different concentrations, the levels of FTH1, GPX4, SLC7A11, SLC3A2, and Nrf2 increased, while ACSL4 levels decreased. Treatment effect was more significant in BH and SP groups compared to BL group (Figures 3A–G). The RT-qPCR data confirmed these tendencies, with similar expression patterns for FTH1, GPX4, SLC7A11, SLC3A2, ACSL4, and Nrf2 across groups (Figures 4A–F). The above data imply that baicalein alters the expression of protein and mRNA of ferroptosis-related pathway genes.

Through RNA-seq analysis, we identified significant differentially expressed genes (DEGs) between the treatment groups (Supplementary: S2-S6). As shown in S2, the Venn diagram clearly illustrates the similarities and differences in gene expression patterns among the control group, baicalein-treated group, and untreated group, with screening criteria set at ≥ 2-fold change and p ≤ 0.05. The results demonstrates that compared to the control group, DSS treatment resulted in significant upregulation of ACSL4 along with marked downregulation of FTH1, SLC7A11, GPX4, and SLC3A2 (see S3A). Notably, as shown in Supplementary Figure S3B, baicalein treatment significantly reversed these DSS-induced expression changes, leading to downregulation of ACSL4 and upregulation of FTH1, SLC7A11, GPX4, and SLC3A2. To further investigate the biological functions of these DEGs, we performed systematic GO enrichment analysis. As illustrated in S4 and S5, gene ontology analysis before and after baicalein treatment revealed significant enrichment of differentially expressed genes in key biological processes including muscle contraction regulation, ion channel function, and synaptic signaling. Particularly noteworthy, KEGG pathway analysis (S5) showed highly significant enrichment of the ferroptosis pathway. These results indicate that baicalein treatment can significantly alter the gene expression profile in DSS-induced ulcerative colitis mice models, with its mechanism of action likely mediated through regulation of ferroptosis-induced cell death pathways.

### 3.4 Baicalein treatment regulates the gut microbiota diversity and structure of DSS-induced UC in mice

**FIGURE 5:**
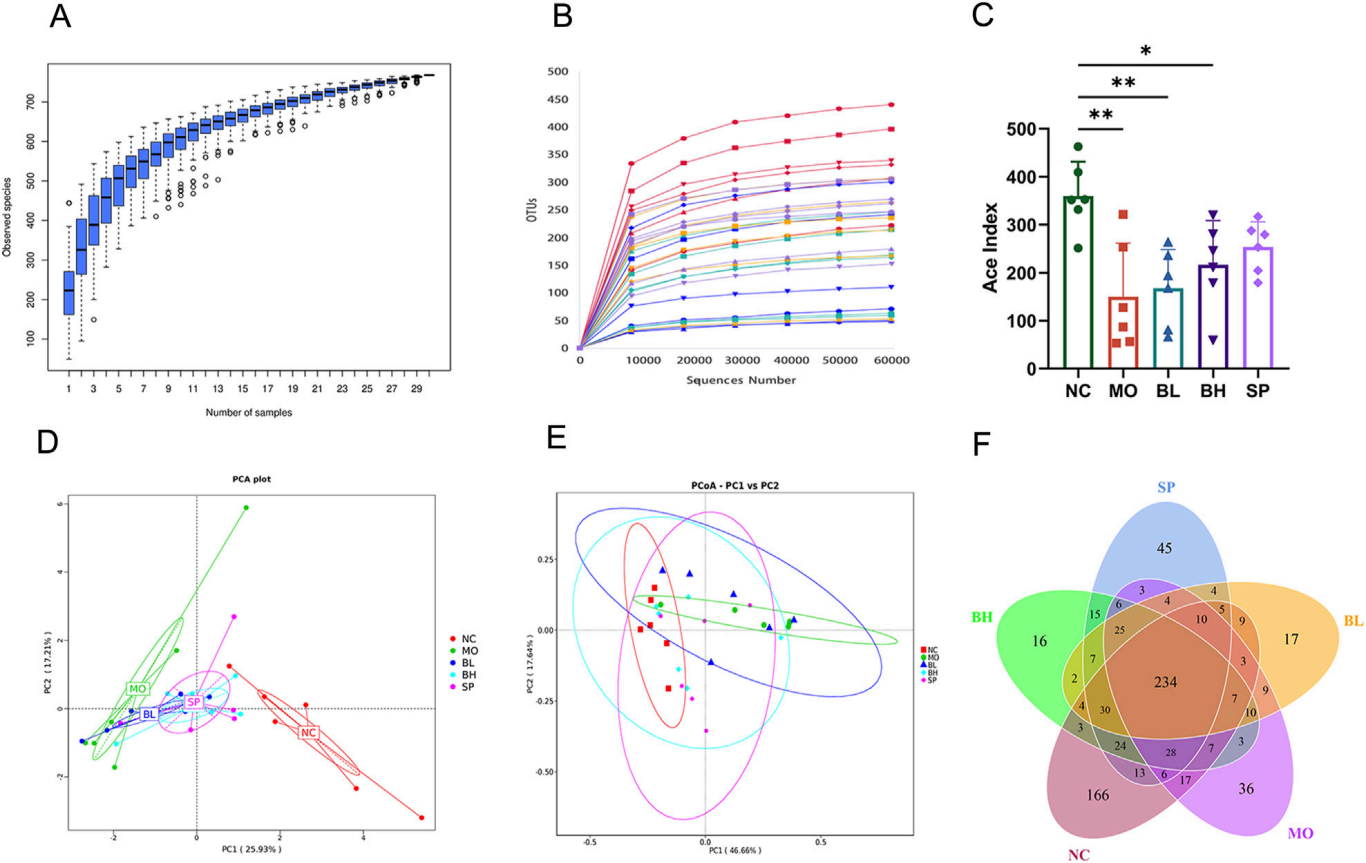
The impact of baicalein on the structure and diversity of gut microbiota in DSS-induced mice. **(A)** Cumulative boxplot of microbiota at OTUs level. **(B)** Dilution curves of microbiota at the OTUs level. **(C)** ACE index of microbiota at the OTUs level. **(D)** PCA analysis (phylum) of microbiota at the OTUs level. **(E)** PCoA analysis based on Weighted Unifrac distance. **(F)** Venn plots (Data were analyzed by one-way ANOVA, with results presented as means ± SD. *N* = 6. ^#^ *p* < 0.05, ^##^ *p* < 0.01, ^###^ *p* < 0.001 vs. NC group; ^*^ *p* < 0.05, ^**^ *p* < 0.01, ^***^ *p* < 0.001 vs. MO group).

We employed the 16S rRNA sequencing method to analyze alterations in intestinal microbiota in UC mice. Dilution curves were created using OTUs clustering to validate sample sequencing data. As sequencing depth increases, the species abundance curve stabilizes between groups, indicating enough sample size and trustworthy of the sequencing data (Figures 5A,B). Firstly, the ACE index was used to assess gut microbiota alpha diversity in the cohort. The MO group had a significantly lower ACE index than NC group. Treatment with baicalein significantly increased the ACE index, while the effect was most prominent in the BH and SP groups (Figure 5C). Similarly, the Chao1 index (reflecting species richness), Shannon index (representing comprehensive diversity), and Simpson index (analyzing dominance) all demonstrated lower values in the DSS group, while varying degrees of recovery were observed following baicalein intervention (S1). These findings are consistent with existing literature reports (e.g., Song et al. (2021)). Secondly, PCA and PCoA analyses conducted at OTUs level demonstrated beta diversity in gut microbiota among the groups. The result showed distinct clustering of intestinal microbial communities among NC, MO, and the treatment groups (BL, BH, and SP). Interestingly, the BH and SP groups of the clustering analysis are closer to the NC group, while the BL group is closer to the MO group (Figures 5D,E). Finally, Venn diagrams showed 234 OTUs shared by the five sample groups. The NC, MO, BL, BH, and SP groups had 166, 36, 17, 16 and 45 OTUs, respectively. These results show that baicalein altered abundance and composition of the intestinal microbiota (Figure 5F).

**FIGURE 6:**
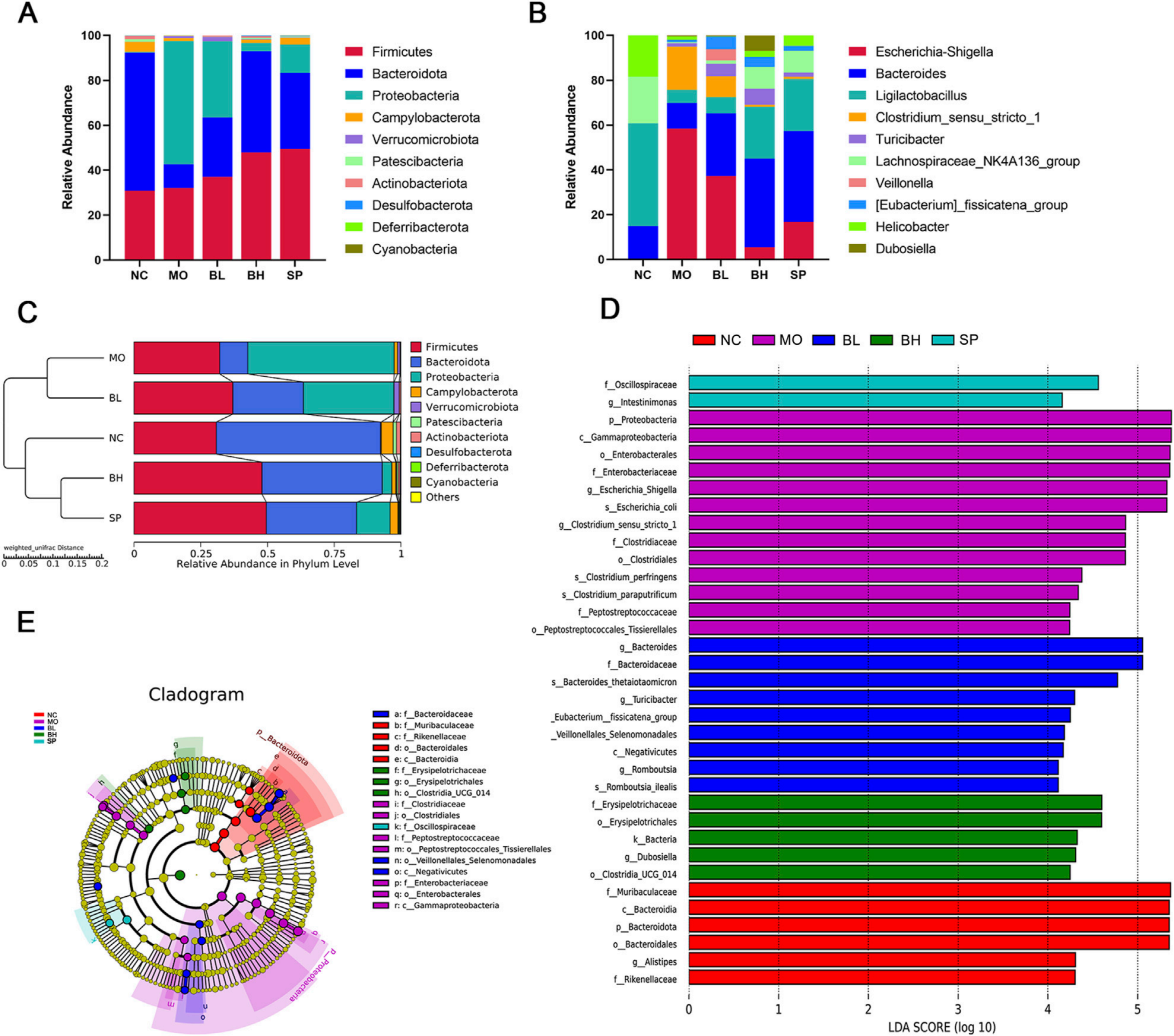
The impact of baicalein on the composition of gut microbiota in DSS-induced mice. **(A)** Species abundance stacked plot at the phylum level. **(B)** Species abundance stacked plot at the genus level. **(C)** Weighted UPGMA dendrogram. **(D)** LDA effect size analysis of the major biomarker taxa. **(E)** LEfSe analysis to determine differences in abundance of different groups. (*N* = 6).

To determine how baicalein affected the composition and organization of the gut microbiota, we examined the phylum and genus alterations in the top 10 species with the highest relative abundance. At the phylum level, *Firmicutes* and *Bacteroidetes* dominated the NC group. The MO group had more *Proteobacteria* and fewer *Bacteroidetes* than the NC group, indicating the increase of the F/B ratio. However, this trend was reversed with the treatment of baicalein (Figure 6A). At the genus level, the top ten microbes by relative abundance included *Escherichia-Shigella*, *Bacteroides*, *Ligilactobacillus*, *Clostridium_sensu_stricto_1*, *Turicibacter*, *Lachnospiraceae_NK4A136_group*, *Veillonella*, *[Eubacterium]_fissicatena_group*, *Helicobacter*, and *Dubosiella*. In addition, *Ligilactobacillus*, *Lachnospiraceae_NK4A136_group*, *Dubosiella*, and *Bacteroides* were the most abundant in the NC group. In contrast, *Ligilactobacillus* (a genus within the *Lachnospiraceae* family) and the *Lachnospiraceae NK4A136 group* exhibited downregulation in the MO group, with subsequent restoration post-intervention. Conversely, *Escherichia coli*, *Shigella*, and *Clostridium_sensu_stricto_1* demonstrated significant upregulation in the MO group. Administration with baicalein treatment and the positive control drug (sulfasalazine) reversed these patterns (Figure 6B). Furthermore, the UPGMA tree analysis showed that the samples from gut microbes in five groups of mice were clustered separately into two groups, while the BH and SP groups showed a closer relationship with the NC group (Figure 6C).

Additionally, we used linear discriminant analysis (LDA) effect sizes (threshold 4) to identify specific bacterial taxa whose abundance was affected by baicalin treatment. Results showed that f_*Muribaculaceae*, c_*Bacteroidia*, p_*Bacteroidota*, o_*Bacteroidales*, g_*Alistipes*, and f_*Rikenellaceae* were enriched in the NC group. p_*Proteobacteria* was significantly enriched in MO group, while g_*Bacteroides* was significantly enriched in BL group. The most enriched bacteria in SP and BH groups were s_*Romboutsia*_*ilealis* and f_*Oscillospiraceae*, respectively. The *cladogram* further demonstrated specific colonic microbial taxa related with baicalein treatment (Figures 6D,E). These results suggest that baicalein significantly alters the composition of the intestinal microbiota.

### 3.5 Relationships among ferroptosis pathways, inflammatory factors, oxidative stress, and intestinal microbiota

**FIGURE 7:**
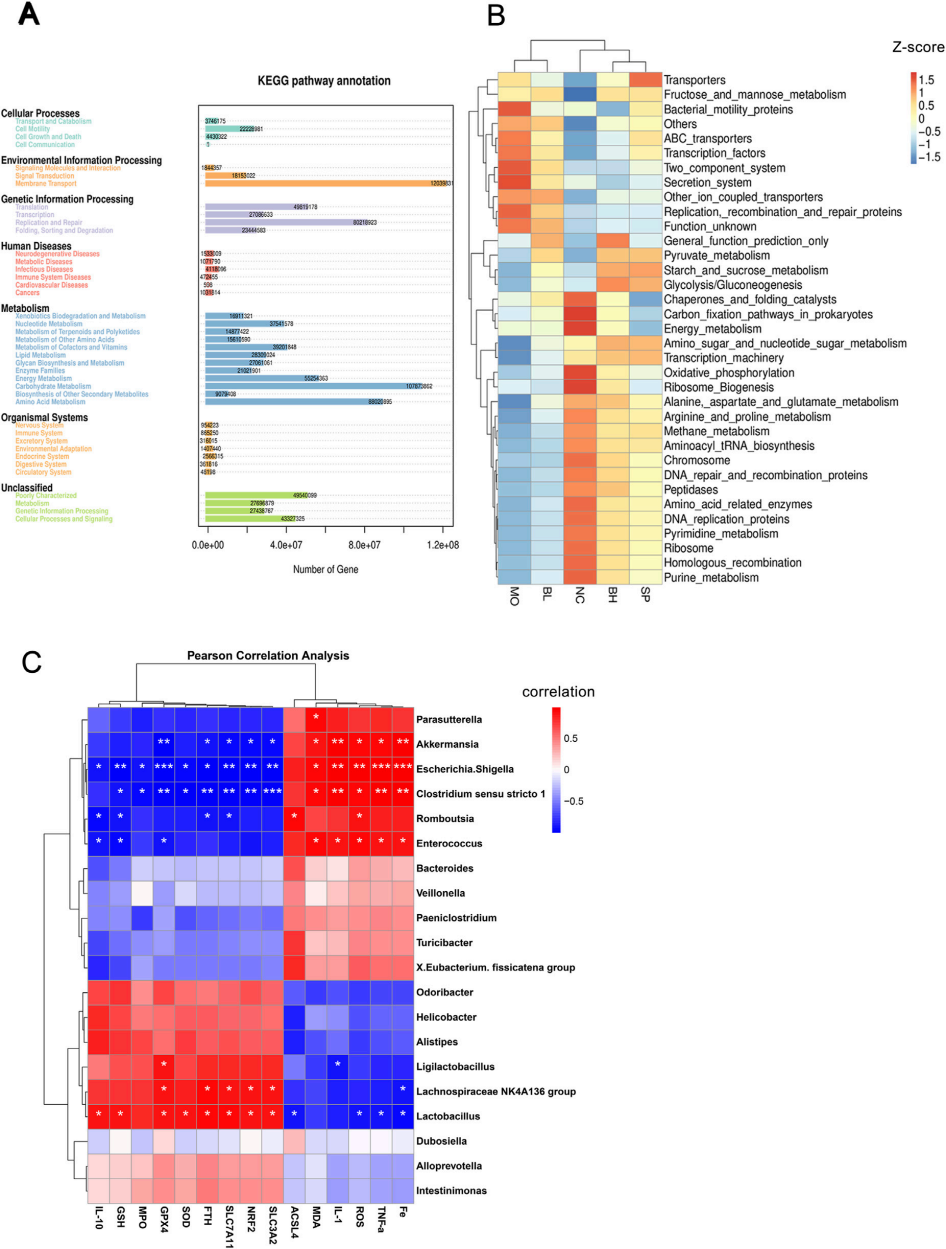
The impact of baicalein on the intestinal microbiota and explores the interrelationships between different indicators and gut flora. **(A)** KEGG gene enrichment analysis. **(B)** Heatmap of KEGG pathway enrichment. **(C)** Pearson correlation analysis between gut microbiota genera and inflammatory factors, oxidative stress markers, and ferroptosis-related proteins. (*N* = 6, ^*^ *p* < 0.05, ^**^ *p* < 0.01, ^***^ *p* < 0.001).

We utilized PICRUSt approach to forecast the functionality using 16S rRNA sequencing and scrutinize functional variances among different groups. Annotation of KEGG pathways unveiled more than 20 million annotated genes, encompassing categories such as cell motility, membrane transport, genetic information processing, and diverse metabolic pathways. These metabolic pathways involved nucleotide, lipid, energy, and amino acid metabolism, potentially linked to ferroptosis (Figures 7A,B).

The twenty most prevalent gut microbial species were examined for correlations with markers of oxidative stress, inflammation, and ferroptosis in UC mice using Pearson’s correlation coefficient. ROS, MDA, MPO, GSH, TNF-α, IL-1β, IL-10, and ferroptosis-related proteins (i.e., FTH1, GPX4, SLC7A11, SLC3A2, Nrf2, ACSL4) were analyzed. In Figure 7C, it could be seen that *Akkermansia*, , *Escherichia-Shigella* and *Clostridium_sensu_stricto_1* showed positive relationships with inflammatory markers (ROS, MDA, TNF-α, and IL-1β), especially IL-1β. These genera showed negative correlations with ferroptosis pathway proteins FTH1, GPX4, SLC7A11, SLC3A2, and Nrf2, with GPX4 showing the significant negative correlation. Additionally, IL-10 exhibited a negative correlation with *Escherichia-Shigella* and a positive correlation with GSH for *Clostridium_sensu_stricto 1*. *Escherichia-Shigella* and *Clostridium_sensu_stricto 1* demonstrated stronger correlations with ferroptosis-related proteins compared to *Akkermansia*. *Lactobacillus* showed negatively correlated with ROS, TNF-α, and ACSL4, but positively correlated with IL-10, GSH, GPX4, Nrf2, SLC7A11, SLC3A2, FTH1, and ACSL4.

## 4 Discussion

Ulcerative colitis (UC) is a chronic IBD primarily affecting the colon and rectum, has a multifactorial effects in intestinal microbiota, and mucosal inflammation (Ordás et al., 2012). Iron-dependent ferroptosis, a form of programmed cell death, is intricately regulated by signaling pathways and cellular metabolic activities (Sun et al., 2023; Yokote et al., 2023). Recent research has identified natural compounds like protocatechuic acid (Yang et al., 2023), astragalus polysaccharides (Chen Y. et al., 2021), dandelion polysaccharides (Yan et al., 2023), and curculigoside (Wang et al., 2020), as potential agents for attenuating UC by inhibiting ferroptosis. Although ferroptosis and its molecular processes are receiving more and more attention in UC, further comprehensive investigations are needed. Our study reveals that baicalein significantly alleviates DSS-induced UC by modulating ferroptosis-related signaling pathways, modifying the intestinal microbiota, and consequently suppressing ferroptosis.

Symptoms of UC include hematochezia, lethargy, diarrhea, and abdominal pain (Feuerstein and Cheifetz, 2014). The main pathological features encompass crypt abscesses, mucosal and submucosal inflammation, as well as structural epithelial such as atrophy, structural disarray, and decreased cupped cell numbers in the intestinal mucosa (Cosnes et al., 2011; Danese and Fiocchi, 2011). In murine models, DSS induces symptoms resembling those seen in UC patients, include hematochezia, weight loss, and colonic shortening (Park et al., 2015). Research has indicated that paclitaxel can enhance colon length, decrease body weight loss, and lower DAI score in DSS-induced colitis in rats (Li W. et al., 2022). Moreover, Herba Origani has been shown to enhance intestinal barrier integrity, alleviate colitis symptoms, reduce colon inflammation, and mitigate histological damage in DSS-induced UC animals animal models (Yu et al., 2023). Consistent with these findings, our research indicated that treating mice with DSS-induced colitis with baicalein resulted in colon lengthening, prevention of weight loss, and restoration of damaged colonic mucosa. The cytokine network significantly influences the immune response and inflammatory signals in ulcerative colitis (Neurath, 2014). TNF-α plays a crucial role by activating mitogen-activated protein kinases (MAPKs) and NF-κB pathways, resulting in the upregulation of pro-inflammatory cytokines and exacerbating intestinal barrier dysfunction in UC patients (Pedersen et al., 2014). Deferasirox pretreatment has been shown to reduce inflammation-related oxidative stress and IL-1β expression in mice with DSS-induced colitis (Wu et al., 2023). IL-10 serves a protective function in the mucosal immune system, and the severity of UC is associated with impaired IL-10 signaling (Glocker et al., 2009). Our findings demonstrate that baicalein significantly upregulates IL-10, an anti-inflammatory cytokine, and simultaneously reduces TNF-α and IL-1β expression. These results indicate that baicalein alleviates epithelial inflammation in UC by regulating the inflammatory cytokine balance, elevating IL-10 levels, and reducing TNF-α and IL-1β expression.

The chronic inflammation in UC is associated with ferroptosis due to inducing lethal iron overload, accumulation of ROS levels, and uncontrolled lipid peroxidation, leading to the demise of intestinal epithelial cells (IECs) (Yokote et al., 2023). Disruptions in the cellular absorption, export, and utilization of iron ions lead to oxidative damage to cells. Transferrin (TF) binds to Fe^3+^ for intracellular transportation. Iron balance is regulated by the conversion of internalized Fe^3+^ to Fe^2+^ by STEAP3, followed by extracellular export through the Ferroportin (FPN) (Namgaladze et al., 2022). Studies have demonstrated that ferritin and its analogs, along with iron chelators, can reduce intracellular iron levels and enhance the expression of FTH1, thereby inhibiting ferroptosis (Feng et al., 2022; Xia et al., 2023). Change of the Fe^2+^ levels and FTH1 in the colon tissue of UC mice were assessed pre- and post-administration of baicalein. Ferritin levels increased with escalating baicalein concentration, while Fe^2+^ levels significantly decreased in the high dose of baicalein group. MDA, GSH, and SOD are antioxidant enzymes present in IECs and serve as markers of inflammation and oxidative stress (Hu et al., 2019). ROS is a significant pathogenic component in ulcerative colitis (Wang et al., 2016), promoting damage to macromolecules such as proteins and DNA. Elevated MDA levels stimulate inflammatory cell activation and oxidative stress (Trivedi and Jena, 2015). Scavenging oxygen-free radicals and controlling ROS levels, SOD protects cells from oxidative damage (Elmaksoud et al., 2021). GSH, a Nrf2-related ROS scavenger, not only converts ROS into stable molecules but also activates GPX4 (Vučković et al., 2021). GPX4 modulates the amino acid antioxidant system via the System Xc-/GSH/GPX4 axis, leading to ferroptosis. In a DSS-induced UC animal model, Wang et al. showed reduced expression of GSH, SOD, and GPX4, alongside elevated levels of iron, ROS, and MDA (Wang et al., 2020). Our biochemical analysis of mouse colon tissue revealed that baicalein-treated groups exhibited lower MDA levels and higher GSH and SOD levels compared to controls (Yang et al., 2023). Furthermore, the baicalein groups exhibited a dose-dependent elevation in the expression of the upstream factor SLC7A11 within the System Xc-/GSH/GPX4 axis, accompanied by elevated levels of GPX4 and GSH (Xu et al., 2022). Nrf2 regulates SLC7A11, influencing GSH metabolism. Studies have shown that EC inhibits Nrf2 regulates activation, leading to reduced SLC7A11 expression and iron accumulation. Additionally, cadherin activation of Nrf2/Hippo/yes-related protein (YAP) promotes ferroptosis by targeting the downstream ferroptosis regulator ACSL4 (Wu et al., 2019). Polyunsaturated fatty acids are believed to be more readily integrated into phospholipids (PLs) in the presence of elevated ACSL4 levels, ultimately resulting in cell death (Zhang et al., 2022). Although ACSL4 expression exhibited a decreasing trend in our current study, Nrf2 and SLC7A11 showed an increasing trend, particularly pronounced in the high-dose baicalein cohorts. These findings suggest an association between baicalein and the ferroptosis pathway, crucial in UC and oxidative stress.

The gut microbiota is closely linked to the pathogenesis of UC through the regulation of ferroptosis. UC is characterized by intestinal barrier disruption, oxidative stress, and dysregulated immune responses. Ferroptosis, an iron-dependent form of cell death driven by lipid peroxidation, may contribute to UC progression through multiple mechanisms (Long et al., 2024). Studies indicate that UC patients often exhibit intestinal iron overload, where aberrant ferritin expression leads to free iron release. This iron catalyzes ROS generation via the Fenton reaction, triggering ferroptosis in intestinal epithelial cells (Chen et al., 2020). Notably, *Lactobacillus* abundance positively correlates with ferritin heavy chain 1 (FTH1), suggesting its potential role in promoting Fe^3+^ storage to mitigate ferroptosis and mucosal damage in UC (Mou et al., 2025). Furthermore, reduced GPX4 expression in UC patients results in glutathione system failure, impairing lipid peroxide clearance. The positive association between *Lactobacillus* and GPX4 supports its protective effects via antioxidant reinforcement (Wang et al., 2023).

Short-chain fatty acids (SCFAs), key metabolites derived from gut microbiota, critically modulate ferroptosis and UC pathology. Produced by Bifidobacterium and *Lactobacillus* through dietary fiber fermentation, SCFAs exert protection by suppressing HIF-2α-mediated upregulation of divalent metal transporter 1 (DMT1), thereby reducing intracellular iron accumulation (Das et al., 2020). Butyrate, a major SCFA, activates the P21/Nrf2 pathway to enhance SOD levels (Kim et al., 2020). Given the impaired Nrf2 function in UC, SCFAs may restore Nrf2-dependent antioxidant responses to counteract ferroptosis. Microbiota-regulated polyunsaturated fatty acid (PUFA) metabolism further influences ferroptosis in UC. Dysbiosis in UC (e.g., increased Prevotella and decreased *Lactobacillus*) may promote ω-6 PUFA enrichment (Chen X. et al., 2021) which is esterified by ACSL4 into oxidation-prone membrane phospholipids. Additionally, microbiota-derived glycodeoxycholic acid exacerbates ferroptosis via the TfR-ACSL4 axis (Liu et al., 2022). Bile acid dysmetabolism in UC patients underscores the significance of the microbiota-bile acid-ferroptosis axis in disease pathogenesis.

While the present study demonstrates positive correlations between *Lactobacillus* and ferroptosis markers (i.e., SLC7A11 and GPX4), further investigation is required to examine differences in microbial metabolites (e.g., SCFAs and bile acids) between UC patients and healthy controls, which would help elucidate the specific mechanisms underlying the microbiota-ferroptosis interaction. Future studies could explore whether supplementation with probiotics (e.g., *Lactobacillus*) or SCFAs can reverse ferroptosis in UC models, thereby providing novel therapeutic strategies for UC treatment. In summary, the gut microbiota influences ferroptosis through iron metabolism regulation, SCFA signaling, and PUFA peroxidation pathways, a mechanism that plays a pivotal role in intestinal epithelial damage and chronic inflammation in UC. Targeting the microbiota-ferroptosis axis (e.g., via probiotic or SCFA supplementation) may represent a promising therapeutic approach for UC.

## 5 Conclusion

Our results revealed that baicalein treatment led to a significant reduction in TNF-αand IL-1β levels, and an increase in IL-10 levels in UC mice. In addition, baicalein decreased MDA and ROS levels while increasing MPO, SOD and GSH levels, indicating its potential anti-inflammatory properties and improved antioxidant capacity in UC mice. Analysis by Western blot and RT-qPCR showed that baicalein treatment upregulated FTH1, GPX4, SLC7A11, SLC3A2, and Nrf2, and downregulated ACSL4 levels, suggesting that the inhibition of ferroptosis pathway in DSS-induced UC mice. In addition, 16S rRNA sequencing showed that baicalein treatment effectively restored the DSS-induced dysbiosis of gut microbiota. This study preliminarily revealed that baicalein alleviates UC by regulating ferroptosis and gut microbiota, though the specific molecular interaction mechanisms require further exploration. Future work will employ multi-omics analysis and gene-editing technologies to focus on the interactions between baicalein and core ferroptosis targets (e.g., GPX4 and ACSL4), as well as the mediating mechanisms of microbiota-derived metabolites. Although the specific mechanisms of certain bacterial species and the ferritin deposition pathway in the pathogenesis of UC need to be further investigated, the potential of baicalein to alleviate UC provides us with new therapeutic strategies.

## Data Availability

The original contributions presented in the study are included in the article/Supplementary Material, further inquiries can be directed to the corresponding author.
