# Supplementary material for: Baicalein ameliorates DSS-induced ulcerative colitis in mice by inhibiting ferroptosis and regulating gut microbiota
Source: Front Pharmacol. 2025 Jul 31;16:1564783. doi: 10.3389/fphar.2025.1564783 (PMC12350334; doi:10.3389/fphar.2025.1564783)
Supplement: Supplementary file 1 [file DataSheet1.docx]

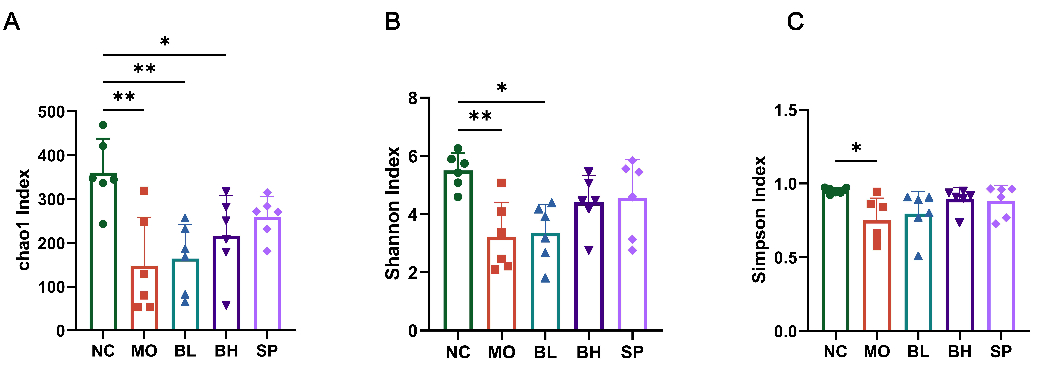


S 1 The impact of baicalein on the structure and diversity of gut microbiota in DSS-induced mice. (A) Chao1 index of microbiota at the OTUs level. (B) Shannon index of microbiota at the OTUs level. (C) Simpson index of microbiota at the OTUs level. (Values were analyzed by one-way ANOVA and results presented as means±SD. n=6. ^#^P<0.05, ^##^P<0.01, ^###^P<0.001 vs. NC group; ^*^P<0.05, ^**^P<0.01, ^***^P<0.001 vs. MO group).


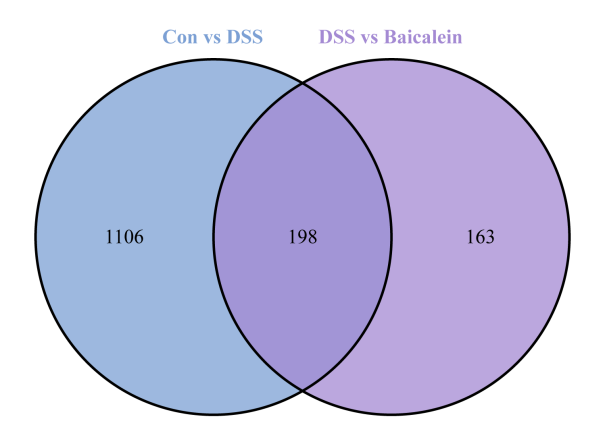


S 2 Venn diagram of differentially expressed genes (DEGs). The diagram illustrates the distribution of DEGs across different comparative groups, where numerically labeled regions indicate the gene counts within each corresponding category. Overlapping areas represent shared DEGs between the respective comparison groups.


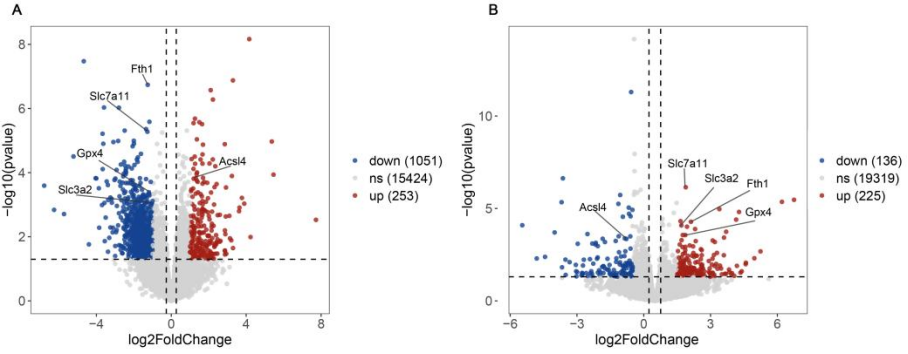


S 3 Volcano plots of differentially expressed genes (DEGs). (A) DSS group versus Control group. (B) Baicalein group versus DSS group. Each data point represents an individual gene, X-axis: Displays the log2-transformed fold change values, quantifying the magnitude of gene expression differences. Y-axis: Represents the negative decadic logarithm of the adjusted p-values, indicating the statistical significance of expression changes. Red points: Significantly upregulated genes (FDR <0.05, fold change ≥2); Blue points: Significantly downregulated genes (FDR <0.05, fold change ≤-2); Black points: Non-significant genes.


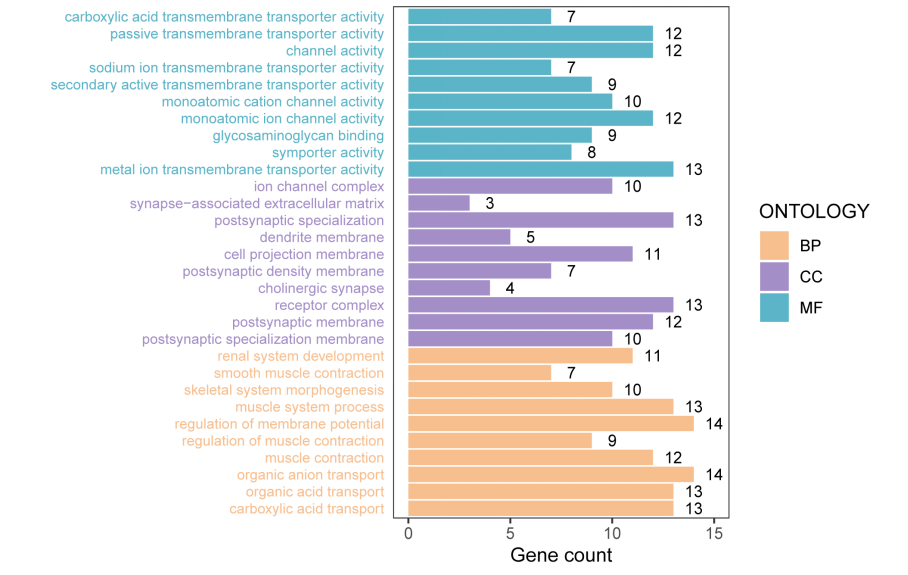


S 4 GO Enrichment Analysis of Differentially Expressed Genes Following Baicalein Treatment in Mice. (Y-axis shows enriched GO biological process terms, and X-axis represents gene counts. BP represents Biological Process; CC represents Cellular Component; MF represents Molecular Function.)


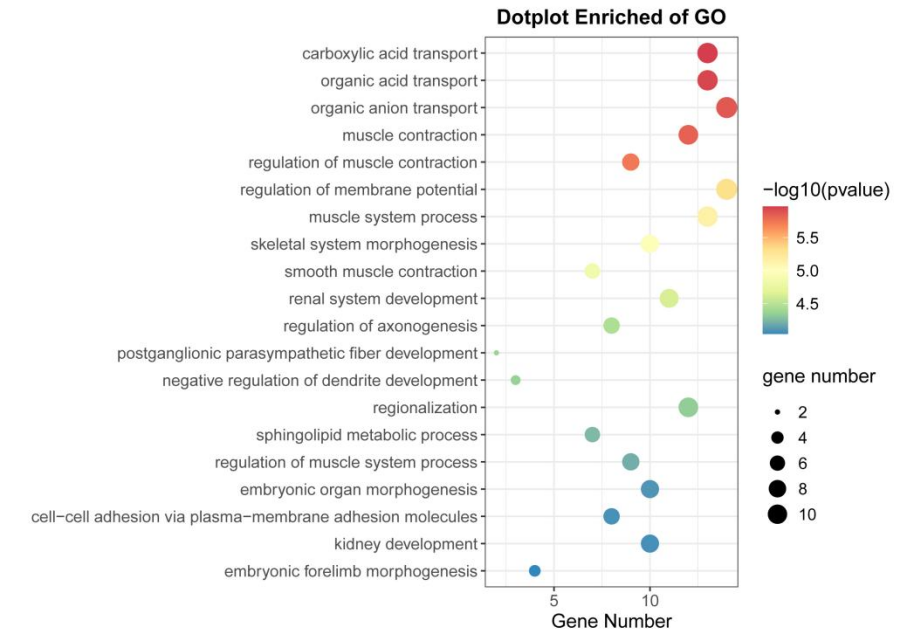


S 5 Dot plot of GO enrichment analysis for differentially expressed genes following baicalein treatment in mice. (X-axis displays enriched GO biological process terms, X-axis indicates the number of differentially expressed genes annotated to each GO term. Dot color gradient (blue to red) represents the significance level of GO term enrichment, with increasing red intensity denoting stronger statistical significance. Dot size corresponds to the number of genes associated with each term.)


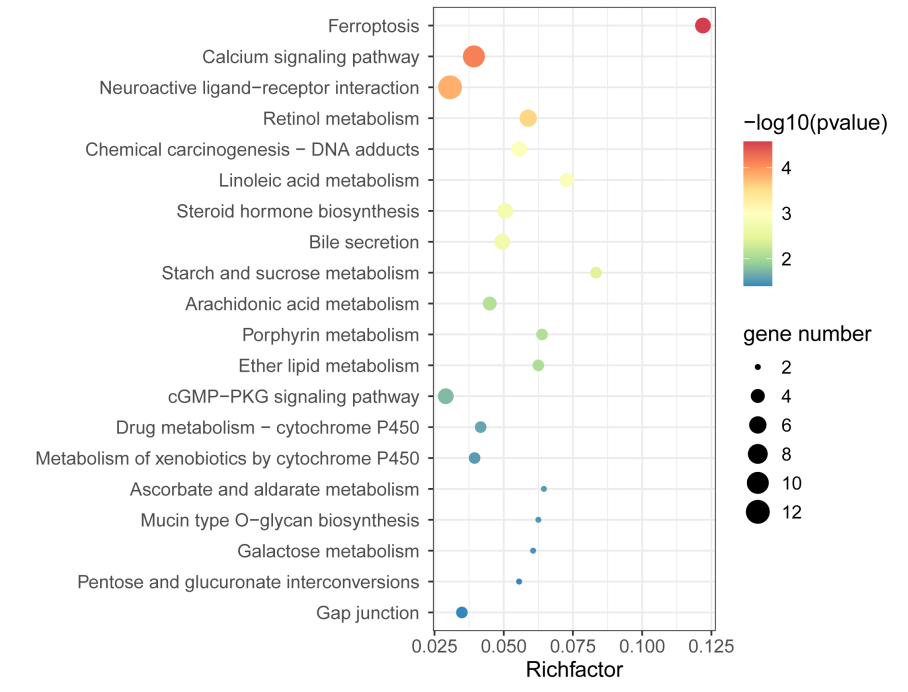


S 6 Dot plot of KEGG enrichment analysis for differentially expressed genes in baicalein-treated mice. (Y-axis shows KEGG metabolic pathway names, X-axis represents the ratio of genes in the pathway to the total annotated genes. Dot color (from blue to red) indicates the significance of pathway enrichment, with redder colors representing stronger significance. Dot size corresponds to the number of differentially expressed genes.)
